# Prediction of hypotension after postural change in robot-assisted laparoscopic prostatectomy using esophageal Doppler monitoring: a prospective observational trial

**DOI:** 10.1038/s41598-021-93990-3

**Published:** 2021-07-16

**Authors:** Na Young Kim, Ki Jun Kim, Tae Lim Kim, Hye Jung Shin, Chaerim Oh, Min Huiy Lee, Ji Young Min, So Yeon Kim

**Affiliations:** 1 Department of Anesthesiology and Pain Medicine, Anesthesia and Pain Research Institute, Yonsei University College of Medicine, 50-1 Yonsei-ro, Seodaemun-gu, Seoul, 03722 Republic of Korea; 2 Biostatistics Collaboration Unit, Yonsei University College of Medicine, Seoul, Republic of Korea; 3 Department of Anesthesiology and Pain Medicine, Eunpyeong St. Mary’s Hospital, College of Medicine, The Catholic University of Korea, 1021 Tongil-ro, Eunpyeong-gu, Seoul, 03312 Republic of Korea

**Keywords:** Medical research, Risk factors

## Abstract

Postural change from a steep Trendelenburg position to a supine position (T-off) during robot-assisted laparoscopic prostatectomy (RALP) induces a considerable abrupt decrease in the mean arterial pressure (MAP). We investigated the variables for predicting postural hypotension induced by T-off using esophageal Doppler monitoring (EDM). One hundred and twenty-five patients undergoing RALP were enrolled. Data on the MAP, heart rate, stroke volume index (SVI), cardiac index, peak velocity, corrected flow time, stroke volume variation, pulse pressure variation, arterial elastance (Ea), and dynamic arterial elastance were collected before T-off and at 1, 3, 5, 7, and 10 min after T-off using EDM. MAP < 60 mmHg within 10 min after T-off was considered to indicate hypotension, and 25 patients developed hypotension. The areas under the curves of the MAP, SVI, and Ea were 0.734 (95% confidence interval [CI] 0.623–0.846; *P* < 0.001), 0.712 (95% CI 0.598–0.825; *P* < 0.001), and 0.760 (95% CI 0.646–0.875; *P* < 0.001), respectively, with threshold values of ≤ 74 mmHg, ≥ 42.5 mL/m^2^, and ≤ 1.08 mmHg/mL, respectively. If patients have MAP < 75 mmHg with SVI ≥ 42.5 mL/m^2^ or Ea ≤ 1.08 mmHg/mL before postural change from T-off during RALP, prompt management for ensuing hypotension should be considered.

Trial registration: NCT03882697 (ClinicalTrial.gov, March 20, 2019).

## Introduction

Robot-assisted laparoscopic prostatectomy (RALP) has become a more favorable surgical technique than open prostatectomy based on postoperative complications, perioperative outcomes, and functional outcomes^1,2^. However, a specific physiologic condition is required for RALP: pneumoperitoneum with carbon dioxide (CO_2_) insufflation and a steep Trendelenburg position of ≥ 30°^3–5^. These combined effects can result in considerable hemodynamic alterations, including > 30% increase in the mean arterial pressure (MAP) and a threefold increase in the central venous pressure (CVP)^3,4^. Furthermore, resuming a supine position with cessation of CO_2_ insufflation afterward induces considerable abrupt decreases in the MAP and CVP^4,5^.

However, data on the prediction of hypotension following a postural change in patients under general anesthesia are limited. Only one study investigated the variables that can predict hypotension after a postural change from the supine to the beach chair position (70° upright position)^6^. However, this hemodynamic change may differ from that in patients undergoing a postural change from a steep Trendelenburg position to a supine position during RALP. Moreover, that previous study only included patients younger than 65 years, with a mean age of approximately 45 years, whose compensatory responses were likely more adequate than those of elderly patients. Elderly patients, especially those with comorbidities, such as hypertension and diabetes mellitus, are susceptible to hypotension after sudden upright postural change because of autonomic system dysfunction and impairment of cardiovascular responses to sudden changes in pathologic states^7,8^. Patients undergoing RALP are mostly elderly (mean age > 60 years) with various comorbidities^2^, which may increase the risk of hypotension when resuming a supine position from a steep Trendelenburg position.

Esophageal Doppler monitoring (EDM) allows for continuous monitoring of cardiac output (CO) and other advanced hemodynamic variables, including preload, afterload, and contractility^9,10^. This technique is a safe, quick, and less invasive method compared with the thermodilution technique with a pulmonary artery catheter^9,10^. Numerous studies have shown that EDM can be a useful supplementary part of standard perioperative hemodynamic monitoring that could reduce postoperative morbidity and length of hospital stay^9^. Therefore, we investigated the variables that could predict postural hypotension induced by changing a patient’s position from a Trendelenburg position to a supine position (T-off) during RALP using EDM.

## Results

### Demographic and intraoperative characteristics

**Figure 1:**
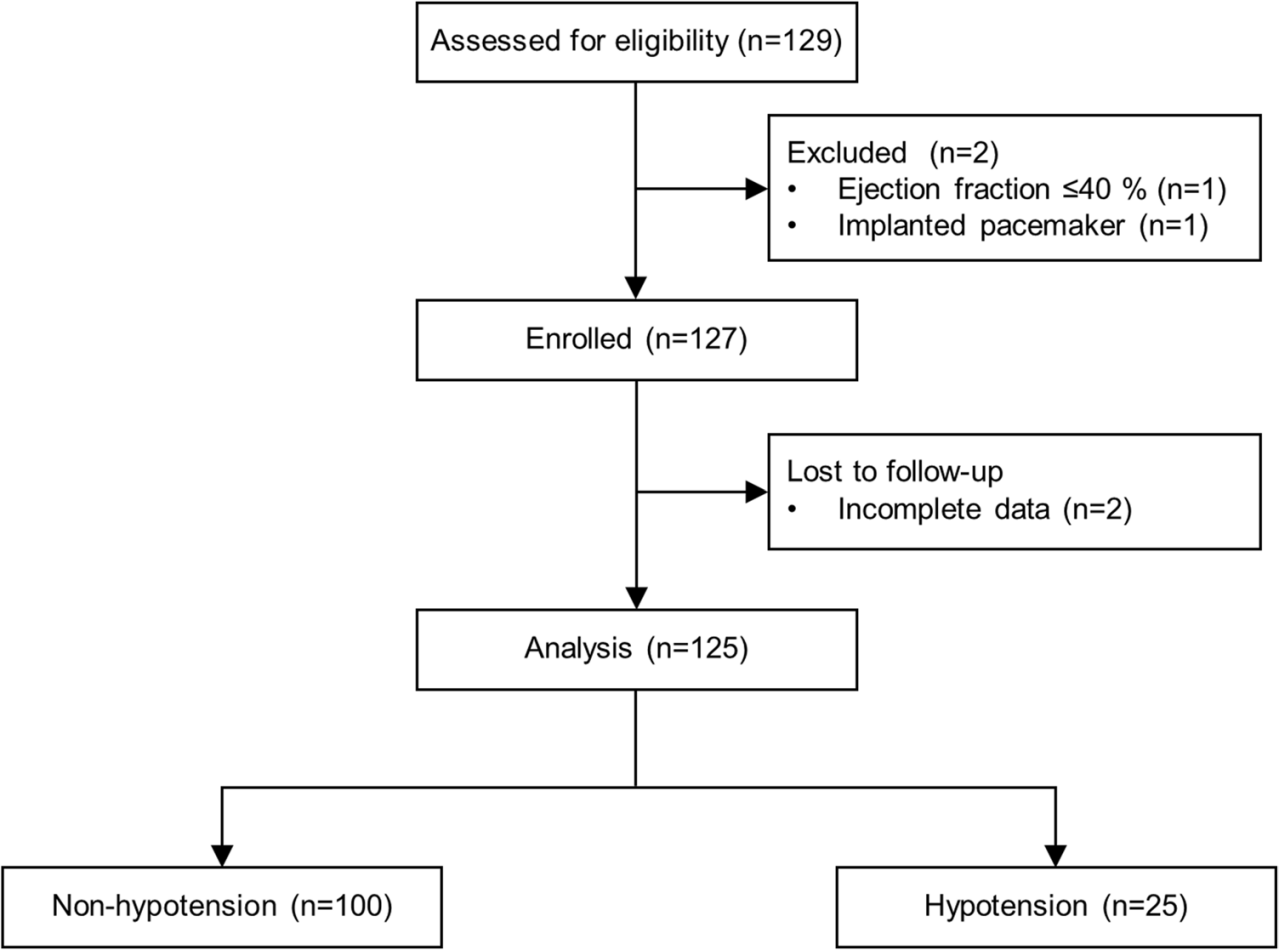
CONSORT flow diagram.

Of the 127 patients enrolled, 2 patients were excluded from the analysis because of incomplete data. Among 125 patients, 25 patients developed hypotension after T-off (Fig. 1). Patients’ characteristics and intraoperative variables are shown in Table 1, and no differences were found between the non-hypotension and hypotension groups, except for the administered dose of ephedrine.

**Table 1:** Patients’ characteristics and intraoperative variables.

	Non-hypotension (n = 100)	Hypotension (n = 25)	*P* value
Age (years)	65.1 ± 6.6	67.6 ± 5.8	0.091
Body mass index (kg/m^2^)	24.9 ± 2.4	23.9 ± 2.3	0.067
**ASA physical status**			0.439
I	19 (19)	4 (16)	
II	59 (59)	18 (72)	
III	22 (22)	3 (12)	
**Comorbidities**			
Hypertension	52 (52)	10 (40)	0.283
Diabetes mellitus	16 (16)	6 (24)	0.382
COPD or asthma	5 (5)	1 (4)	> 0.999
Coronary artery disease	1 (1)	1 (4)	0.361
Cerebrovascular disease	2 (2)	1 (4)	0.559
**Intraoperative variables**			
Tidal volume (mL)	510 [480–530]	520 [480–540]	0.614
Fluid intake until T-off (mL)	850 [700–1050]	850 [700–900]	0.628
Blood loss until T-off (mL)	300 [150–500]	200 [100–400]	0.233
Administered dose of ephedrine (mg)			
During 10 min after T-off	0	4 [4–4]	< 0.001
During the entire operation	0 [0–4]	8 [4–12]	< 0.001

Values are presented as mean ± standard deviation, number of patients (%), or median [interquartile range].

*ASA* American Society of Anesthesiologists; *COPD* chronic obstructive pulmonary disease; *T-off* postural change from the Trendelenburg position to the supine position.

### Hemodynamic variables

Changes in hemodynamic variables are presented in Table 2. Before T-off, the MAP was significantly lower in the hypotension group than in the non-hypotension group (Bonferroni-corrected *P* = 0.027). The MAP significantly decreased in both groups after T-off; however, the changes in the MAP over time were significantly different between the groups (*P*_Group×Time_ < 0.001). The stroke volume index (SVI) was significantly higher (Bonferroni-corrected *P* = 0.004), whereas arterial elastance (Ea) was significantly lower in the hypotension group (Bonferroni-corrected *P* = 0.005) than in the non-hypotension group before T-off. During 10 min after T-off, the hypotension group maintained significantly higher SVI values and lower Ea values than the non-hypotension group. The heart rate (HR), cardiac index (CI), peak velocity (PV), corrected flow time (FTc), stroke volume variation (SVV), pulse pressure variation (PPV), and dynamic arterial elastance (Eadyn) were comparable between the groups before and after T-off, except for a significantly higher PV value at 10 min after T-off in the hypotension group than in the non-hypotension group (Bonferroni-corrected *P* = 0.025).

**Table 2:** Changes in hemodynamic variables.

	Before T-off	T-off 1 min	T-off 3 min	T-off 5 min	T-off 7 min	T-off 10 min	*P*_Group×Time_
**MAP (mmHg)**							< 0.001
Non-hypotension	81 ± 10	79 ± 11^†^	80 ± 12	78 ± 12^†^	75 ± 12^†^	76 ± 12^†^	
Hypotension	74 ± 8*	63 ± 8*^,†^	62 ± 6*^,†^	64 ± 7*^,†^	64 ± 8*^,†^	63 ± 7*^,†^	
**HR (beats/min)**							0.037
Non-hypotension	70 ± 9	72 ± 10^†^	71 ± 10^†^	70 ± 10	70 ± 10	71 ± 10	
Hypotension	68 ± 9	67 ± 8	67 ± 8	67 ± 8	67 ± 9	68 ± 9	
**SVI (mL/m**^**2**^**)**							0.009
Non-hypotension	42.9 ± 9.8	40.5 ± 9.9^†^	40.9 ± 10.2^†^	41.6 ± 10.4^†^	43.0 ± 10.9	42.9 ± 10.7	
Hypotension	50.9 ± 11.0*	46.5 ± 11.1^†^	48.3 ± 10.5*^,†^	49.8 ± 10.9*	51.0 ± 11.9*	52.4 ± 11.5*	
**CI (L/min/m**^**2**^**)**							0.001
Non-hypotension	3.0 ± 0.8	2.9 ± 0.8	2.9 ± 0.9^†^	2.9 ± 0.9	3.0 ± 0.9	3.0 ± 0.9	
Hypotension	3.4 ± 0.6	3.1 ± 0.7^†^	3.2 ± 0.7^†^	3.3 ± 0.7	3.4 ± 0.7	3.5 ± 0.7	
**PV (cm/s)**							0.136
Non-hypotension	58 ± 11	55 ± 12^†^	55 ± 11^†^	57 ± 12^†^	58 ± 13	58 ± 13	
Hypotension	64 ± 15	61 ± 16^†^	61 ± 14^†^	63 ± 14	65 ± 15	66 ± 15*	
**FTc (ms)**							0.003
Non-hypotension	340 ± 27	346 ± 32^†^	345 ± 32	344 ± 31	349 ± 29^†^	351 ± 31^†^	
Hypotension	354 ± 22	342 ± 24^†^	351 ± 23	349 ± 27	353 ± 28	362 ± 26	
**SVV (%)**							0.339
Non-hypotension	12 ± 4	13 ± 9	13 ± 8	12 ± 9	12 ± 8	12 ± 9	
Hypotension	13 ± 5	17 ± 13	15 ± 9	13 ± 7	13 ± 8	14 ± 9	
**PPV (%)**							0.633
Non-hypotension	8 ± 3	6 ± 3^†^	6 ± 3^†^	6 ± 2^†^	6 ± 3^†^	6 ± 3^†^	
Hypotension	8 ± 4	7 ± 4	7 ± 3	6 ± 2^†^	6 ± 4	6 ± 2^†^	
**Ea (mmHg/mL)**							0.087
Non-hypotension	1.40 ± 0.37	1.40 ± 0.39	1.47 ± 0.48^†^	1.44 ± 0.46	1.41 ± 0.47	1.40 ± 0.46	
Hypotension	1.09 ± 0.31*	0.98 ± 0.28*	1.02 ± 0.31*	1.03 ± 0.26*	0.99 ± 0.28*	1.00 ± 0.27*	
**Eadyn**							0.979
Non-hypotension	1.35 ± 0.40	1.12 ± 0.47^†^	1.08 ± 0.57^†^	1.18 ± 0.51	1.19 ± 0.50	1.21 ± 0.51	
Hypotension	1.27 ± 0.47	1.07 ± 0.48	1.09 ± 0.44	1.12 ± 0.39	1.08 ± 0.41	1.20 ± 0.42	

Values are presented as mean ± standard deviation. *T-off* positional change from the Trendelenburg position to the supine position; *MAP* mean arterial pressure; *HR* heart rate; *SVI* stroke volume index; *CI* cardiac index; *PV* peak velocity; *FTc* corrected flow time; *SVV* stroke volume variation; *PPV* pulse pressure variation; *Ea* arterial elastance, calculated as (systolic arterial pressure × 0.9)/stroke volume; *Eadyn* dynamic arterial elastance, calculated as SVV/PPV.

*P*_Group×Time_, *P* value of the group and time interaction obtained by linear mixed model analysis.

*Bonferroni-corrected *P* < 0.05 compared with the non-hypotension group.

^†^Bonferroni-corrected *P* < 0.05 compared with the value of “before T-off” in each group.

### Prediction of hypotension

**Figure 2:**
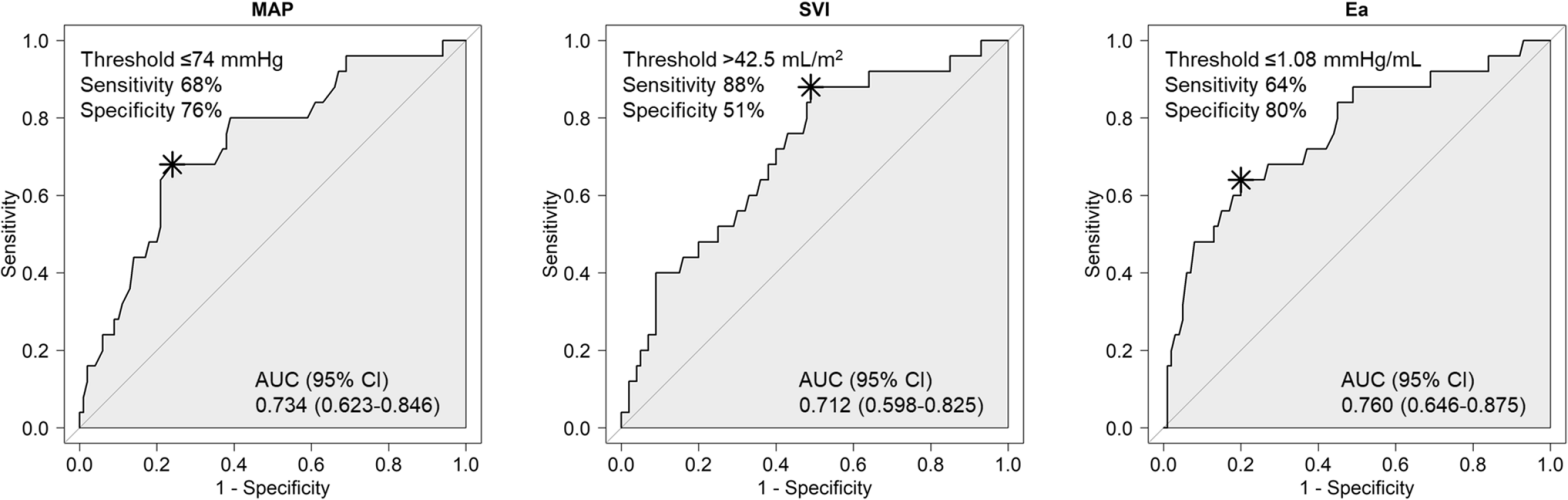
Areas under the receiver operating characteristics curves (AUCs) of each index for predicting hypotension after postural change from the Trendelenburg position to the supine position. *CI* confidence interval; *MAP* mean arterial pressure; *SVI* stroke volume index; *Ea* arterial elastance.

Areas under the receiver operating characteristic curves (AUCs) of each index for predicting hypotension after T-off are presented in Table 3. Among the variables, the MAP, SVI, and Ea showed AUCs > 0.7 (AUC 0.734; 95% confidence interval [CI] 0.623–0.846; *P* < 0.001; AUC 0.712; 95% CI 0.598–0.825; *P* < 0.001; AUC 0.760; 95% CI 0.646–0.875; *P* < 0.001, respectively). The optimal threshold values of the MAP, SVI, and Ea were 74 mmHg (sensitivity 68%, specificity 76%), 42.5 mL/m^2^ (sensitivity 88%, specificity 51%), and 1.08 mmHg/mL (sensitivity 64%, specificity 80%), respectively (Fig. 2).

**Table 3:** Areas under the receiver operating characteristics curves of each index for predicting hypotension after postural change from the Trendelenburg position to the supine position.

	Area under the curve	95% confidence interval	*P* value
MAP (mmHg)	0.734	0.623–0.846	< 0.001
HR (beats/min)	0.518	0.427–0.608	0.797
SVI (mL/m^2^)	0.712	0.598–0.825	< 0.001
CI (L/min/m^2^)	0.689	0.581–0.797	< 0.001
PV (cm/s)	0.609	0.476–0.743	0.108
FTc (ms)	0.656	0.545–0.767	0.006
SVV (%)	0.522	0.426–0.617	0.764
PPV (%)	0.523	0.381–0.665	0.748
Ea (mmHg/mL)	0.760	0.646–0.875	< 0.001
Eadyn	0.558	0.426–0.689	0.386

*MAP* mean arterial pressure; *HR* heart rate; *SVI* strove volume index; *CI* cardiac index; *PV* peak velocity; *FTc* corrected flow time; *SVV* stroke volume variation; *PPV* pulse pressure variation; *Ea* arterial elastance, calculated as (systolic arterial pressure × 0.9)/stroke volume; *Eadyn* dynamic arterial elastance, calculated as SVV/PPV.

## Discussion

This prospective observational study was the first to investigate the hemodynamic variables for predicting hypotension after T-off during RALP. With the use of EDM, we found that a high SVI (threshold of 42.5 mL/m^2^) and low Ea (threshold of 1.08 mmHg/mL) can be used to predict postural hypotension during RALP.

Laparoscopic surgery with CO_2_ insufflation produces considerable hemodynamic changes^11^. In addition to CO_2_ pneumoperitoneum, the steep Trendelenburg position is also used during RALP; these conditions result in more pronounced hemodynamic changes^3–5^. The Trendelenburg position induces an increase in the MAP, whereas resuming the supine position leads to a decrease in the MAP^3–5^. In our study, 20% of patients developed hypotension (MAP < 60 mmHg) within 10 min after resuming the supine position, and considering the patients’ age (mean age: approximately 65 years) and comorbidities (> 80% of patients with comorbidities), this percentage is enough to warrant attention. According to a recent systematic review, there were moderately or highly elevated risks of any end-organ injury after non-cardiac surgery when patients had MAP < 65–60 mmHg for ≥ 5 min or MAP < 55–50 mmHg for any duration^12^. In the present study, 3 of 25 patients developed MAP < 55 mmHg, despite the administration of ephedrine when the MAP was < 60 mmHg (data not shown). Therefore, predicting postural hypotension may be crucial for administering prompt management as needed, especially in elderly patients with comorbidities.

Because of their limited physiologic reserve, elderly people are more susceptible to pathologic conditions. Likewise, orthostatic hypotension, a decrease in blood pressure when standing up, is commonly seen in the elderly population^7,8^. The physiologic condition when resuming a supine position suddenly after a prolonged Trendelenburg position may be similar to that with a sudden change to the upright position. Orthostatic intolerance in elderly individuals is associated with age-related impairment in baroreflex-mediated vasoconstriction and an increase in the HR along with medical conditions such as hypertension and diabetes mellitus^7,8^. Moreover, older age, pre-induction high blood pressure, and the presence of diabetes mellitus were associated with an increased risk of general anesthesia induction-related hypotension^13^. Therefore, we anticipated that the patients developing postural hypotension would be older and with more comorbidities. However, the mean age and incidence of hypertension or diabetes mellitus were not different between the patients who developed hypotension and those who did not (Table 1). As our study focused on the hemodynamic variables and not on patients’ characteristics for the prediction of postural hypotension, further studies are needed to make a definite conclusion on the association of aging or comorbidities with postural hypotension in patients under general anesthesia.

EDM offers minimally invasive continuous real-time hemodynamic monitoring with an extremely low incidence of complications^9,10^. The two commonly used EDM systems are CardioQ (Deltex Medical, Chichester, UK) and Hemosonic (Arrow International, Cleveland, OH, USA)^9,10^. The probe of CardioQ is 6 mm in diameter, which is smaller than that of Hemosonic and approximately the same as that of a nasogastric tube^10^. Along with the small diameter, the probe has an internal spring coil that provides an optimal balance between flexibility and rigidity for easier insertion^10^. The CardioQ probe emits a 4-MHz continuous-wave ultrasound signal at a fixed angle of 45° and measures descending aortic blood flow velocity at the midthoracic level (between the fifth and sixth thoracic vertebrae), at which the aorta and esophagus run approximately parallel^9,10^.

Monitoring of CO itself may be insufficient for therapeutic decision-making. For example, a low CO level can result from hypovolemia, increased systemic vascular resistance (SVR), and decreased contractility. With the use of CardioQ, assessment of preload, afterload, and contractility is possible with the values of FTc and PV^9,10^. A low FTc with a normal PV, a low FTc with a low PV, and a normal FTc with a low PV may indicate low preload, increased afterload, and decreased contractility, respectively^9,10^. The values of FTc and PV before T-off were not different between the non-hypotension and hypotension groups (Table 2), resulting in the AUC for the prediction of postural hypotension being < 0.7 (Table 3). In addition, the dynamic preload indices SVV and PPV and their ratio (SVV/PPV), also known as Eadyn, which predict the arterial pressure response following volume expansion^14^, failed to predict postural hypotension (Table 3). In a previous study investigating hemodynamic variables for the prediction of hypotension in the beach chair position, SVV predicted postural hypotension with AUC 0.691 (95% CI 0.525–0.857; *P* = 0.046)^6^. However, FTc, SVV, and PPV, which are known to be closely related to preload, did not predict postural hypotension induced by T-off in our study.

An SVI value before T-off was found to be a predictor of postural hypotension after T-off. Contrary to a previous result showing that a low SVI (≤ 41.8 mL/m^2^) was a predictor of hypotension in the beach chair position (AUC 0.787; 95% CI 0.647–0.926; *P* = 0.003)^6^, a high SVI (≥ 42.5 mL/m^2^) was a predictor of hypotension after resuming the supine position. Meanwhile, Ea ≤ 1.08 mmHg/mL before T-off was also revealed as a predictor of hypotension. Ea is the ratio of the left ventricular end-systolic pressure to the stroke volume (SV)^15^, and Ea is calculated by the equation (systolic arterial pressure × 0.9)/SV in CardioQ. Therefore, the finding that a low Ea being a predictor is in concordance with a high SVI being a predictor because Ea is inversely related to SV. Ea reflects arterial tone variation and is an expression of the complex association of different arterial properties, such as wall stiffness, compliance, and outflow resistance^15^. Ea has a strong correlation with SVR and is inversely related to compliance; Ea decreases with a lower SVR and higher compliance^16,17^. Therefore, we can deduce that patients with reduced Ea, in other words, those who have a more compliant artery, developed postural hypotension. In a previous study investigating etomidate-induced hypotension, patients who developed hypotension showed a decrease in Ea with an increase in arterial compliance after etomidate injection, whereas patients who did not develop hypotension showed no changes in these values^17^. When a 50-μg bolus of phenylephrine was compared with a 5-μg bolus of norepinephrine in patients with general anesthesia-induced hypotension, phenylephrine led to an increase in the MAP with a larger decrease in the SV and arterial compliance than norepinephrine^18^. In our study, MAP ≤ 74 mmHg before T-off was also a predictor of postural hypotension after T-off. Therefore, a bolus of phenylephrine can be considered if patients have MAP < 75 mmHg before a postural change because phenylephrine can increase the MAP along with decreasing the SV and arterial compliance (i.e., an increase in the Ea). However, further studies are required to determine the beneficial effect of the prophylactic administration of a vasopressor on the prevention of postural hypotension.

The present study has some limitations. First, we used only one definition of hypotension (MAP < 60 mmHg). Previous studies used various definitions of intraoperative hypotension, including a threshold based on the absolute blood pressure or relative blood pressure (a percentage-wise or absolute decrease from the baseline blood pressure)^12^. Therefore, threshold values of SVI and Ea for the prediction of hypotension can be changed according to the definition of hypotension. However, MAP < 60 mmHg may be appropriate for defining postural hypotension because MAP < 60 mmHg was the most commonly used definition of intraoperative hypotension in previous studies^6,12^. Notably, an MAP target of 60–65 mmHg did not increase the 90-day mortality in critically ill elderly patients (≥ 65 years) compared with those in the usual care group^19^. Secondly, all patients in our study were placed in a constant setting of 30° Trendelenburg position with pneumoperitoneum at the pressure of 12 mmHg. Therefore, the effects of different settings of head down angle and intra-abdominal pressure on postural hypotension after T-off was not explored. In addition, since T-off with cessation of CO_2_ insufflation was done in a routine, concurrent manner at our center, each individual effect of postural change and cessation of insufflation could not be determined. Therefore, further studies focusing on the effects of postural change and insufflation pressure separately are needed for more detailed conclusions. Lastly, we collected the data within a limited time interval (from before T-off to 10 min after T-off); therefore, we could not determine the occurrence of post-induction hypotension, which may be correlated with postural hypotension. However, this is beyond the scope of our study because we focused only on hemodynamic variables for predicting postural hypotension.

In conclusion, EDM with CardioQ can be used to predict postural hypotension induced by T-off during RALP. The high SVI and low Ea values before postural change can be used to predict the occurrence of postural hypotension. Prompt management for ensuing hypotension should be considered if patients have MAP < 75 mmHg with SVI ≥ 42.5 mL/m^2^ or Ea ≤ 1.08 mmHg/mL before a postural change.

## Methods

### Study design and patient population

This prospective observational trial was approved by the Institutional Review Board and Hospital Research Ethics Committee (Yonsei University Health System, Seoul, Korea; protocol no. 4–2018-0976) and was performed in accordance with relevant guidelines and regulations. Between March 2019 and December 2020, 127 patients with an American Society of Anesthesiologists physical status of I–III, aged ≥ 20 years, and with prostate cancer who were scheduled for RALP were enrolled after written informed consent was obtained from each patient. Patients were excluded if they had left ventricular ejection fraction < 40%, pre-existing severe vascular disease, implanted pacemaker, autonomic nervous system impairment, or a history of esophageal varices.

### Anesthesia and hemodynamic monitoring

After arrival in the operating room, the patients were monitored with electrocardiography, and data on the peripheral oxygen saturation, non-invasive blood pressure, and Patient State Index (PSI) were collected using a SedLine® electroencephalograph sensor (Masimo Corp., Irvine, CA, USA). Following intravenous administration of 0.1 mg of glycopyrrolate as a premedication, anesthesia was induced with a bolus of propofol (1.5–2 mg/kg) and remifentanil (0.5 µg/kg) and maintained with sevoflurane (0.8–1.0 age-adjusted minimal alveolar concentration in 50% O_2_/air) and remifentanil (0.02–0.1 µg/kg/min) by targeting a PSI of 25–50. Neuromuscular blockade was achieved with the administration of 1.2 mg/kg of rocuronium, and its depth was maintained at 0–2 train-of-four by continuous infusion of rocuronium during pneumoperitoneum. Mechanical ventilation was delivered with an air–oxygen mixture (fraction of inspired oxygen = 0.4) at a tidal volume of 7–8 mL/kg and a positive end-expiratory pressure of 5 cmH_2_O. Following cannulation of the radical artery, the probe of EDM was inserted, and CO was continuously monitored. A single surgeon performed all RALPs. When pneumoperitoneum was induced by CO_2_ insufflation, the patients were placed in a 30° Trendelenburg position, and the intra-abdominal pressure was maintained at 12 mmHg. With cessation of CO_2_ insufflation_,_ T-off was performed. Hypotension, defined as MAP < 60 mmHg, was recorded during 10 min after T-off and treated with a bolus of ephedrine in 4-mg increments.

### Data collection

Data on the following variables were collected with EDM using CardioQ before T-off and at 1, 3, 5, 7, and 10 min after T-off: the MAP, HR, SVI, CI, PV, FTc, SVV, PPV, Ea which was calculated as (systolic arterial pressure × 0.9)/SV, Eadyn which was calculated as SVV/PPV. The values of SVI, CI, PV, and FTc were obtained in the flow monitoring mode, whereas PPV, Ea, and Eadyn were obtained in the pressure monitoring mode. All variables before T-off were derived from the average of three consecutive measurements every 30 s and last measurement was done 30 s before T-off. The position of the probe was continuously monitored and adjusted for the best signal for descending aorta.

### Statistical analysis

In a preliminary study with 50 patients, the incidence of hypotension was 12% after T-off. A sample size of 125 patients achieved a power of 90%, which allowed us to demonstrate a difference of 0.25 in the AUC under the null hypothesis of 0.5 and an AUC under the alternative hypothesis of 0.75 using a two-sided z test at a significance level of 0.05. To evaluate the ability of variables to predict hypotension after a postural change, quantification was performed by calculating the AUCs of the hypotension group using the DeLong method. After the receiver-operating characteristic curve was constructed, the optimal threshold value was determined by choosing the maximum value of the Youden index, which was calculated as sensitivity + specificity − 1. All values are presented as mean ± standard deviation, median [interquartile range], or number of patients (proportion). Close-to-normal continuous variables were analyzed using an independent t-test, and the other continuous variables were analyzed using a Mann–Whitney U test. Categorical variables were analyzed by the chi-square test or Fisher exact test as appropriate. For repeated-measure variables, a linear mixed model analysis was performed to determine group and time effects, and a compound symmetry covariance structure was used for the within-subject effect. To adjust for multiple comparisons for post-hoc analysis, the Bonferroni correction was applied. A *P* value of < 0.05 was considered to be significant. All analyses were conducted using SAS version 9.4 (SAS Institute Inc., Cary, NC, USA).

## References

[1] Novara G (2012). Systematic review and meta-analysis of perioperative outcomes and complications after robot-assisted radical prostatectomy. Eur. Urol..

[2] Leow JJ (2016). Robot-assisted versus open radical prostatectomy: A contemporary analysis of an all-payer discharge database. Eur. Urol..

[3] Lestar M, Gunnarsson L, Lagerstrand L, Wiklund P, Odeberg-Wernerman S (2011). Hemodynamic perturbations during robot-assisted laparoscopic radical prostatectomy in 45° Trendelenburg position. Anesth. Analg..

[4] Rosendal C, Markin S, Hien MD, Motsch J, Roggenbach J (2014). Cardiac and hemodynamic consequences during capnoperitoneum and steep Trendelenburg positioning: lessons learned from robot-assisted laparoscopic prostatectomy. J. Clin. Anesth..

[5] Pawlik MT (2020). Pronounced haemodynamic changes during and after robotic-assisted laparoscopic prostatectomy: a prospective observational study. BMJ Open.

[6] Jo YY, Jung WS, Kim HS, Chang YJ, Kwak HJ (2014). Prediction of hypotension in the beach chair position during shoulder arthroscopy using pre-operative hemodynamic variables. J. Clin. Monit. Comput..

[7] Goswami N, Blaber AP, Hinghofer-Szalkay H, Montani J-P (2017). Orthostatic intolerance in older persons: Etiology and countermeasures. Front. Physiol..

[8] Uchmanowicz I, Chudiak A, Jankowska-Polańska B, Gobbens R (2017). Hypertension and frailty syndrome in old age: Current perspectives. Card. Fail. Rev..

[9] Schober P, Loer SA, Schwarte LA (2009). Perioperative hemodynamic monitoring with transesophageal Doppler technology. Anesth. Analg..

[10] King SL, Lim MS (2004). The use of the oesophageal Doppler monitor in the intensive care unit. Crit. Care Resusc..

[11] Atkinson TM, Giraud GD, Togioka BM, Jones DB, Cigarroa JE (2017). Cardiovascular and ventilatory consequences of laparoscopic surgery. Circulation.

[12] Wesselink EM, Kappen TH, Torn HM, Slooter AJC, van Klei WA (2018). Intraoperative hypotension and the risk of postoperative adverse outcomes: a systematic review. Br. J. Anaesth..

[13] Jor O (2018). Hypotension after induction of general anesthesia: occurrence, risk factors, and therapy. A prospective multicentre observational study. J. Anesth..

[14] García MIM, Cano AG, Romero MG (2011). Dynamic arterial elastance to predict arterial pressure response to volume loading in preload-dependent patients. Crit. Care.

[15] Guarracino F, Baldassarri R, Pinsky MR (2013). Ventriculo-arterial decoupling in acutely altered hemodynamic states. Crit. Care.

[16] Bond O (2019). Relationship between microcirculatory perfusion and arterial elastance: A pilot study. Crit. Care Res. Pract..

[17] Abou Arab O (2019). Etomidate-induced hypotension: A pathophysiological approach using arterial elastance. Anaesth. Crit. Care Pain Med..

[18] Vallée F (2017). Norepinephrine reduces arterial compliance less than phenylephrine when treating general anesthesia-induced arterial hypotension. Acta Anaesthesiol. Scand..

[19] Lamontagne F (2020). Effect of reduced exposure to vasopressors on 90-day mortality in older critically ill patients with vasodilatory hypotension: A randomized clinical trial. JAMA.

